# The impact of healthcare industry convergence on the performance of the public health system: a geospatial modeling study of provincial panel data from China

**DOI:** 10.3389/fpubh.2023.1194375

**Published:** 2023-09-12

**Authors:** Wei Li, Ke Zhu, Echu Liu, Wuzhen Peng, Cheng Fang, Qiong Hu, Limei Tao

**Affiliations:** ^1^Department of Data Science, Dongfang College, Zhejiang University of Finance and Economics, Haining, China; ^2^School of Data Science, Zhejiang University of Finance and Economics, Hangzhou, China; ^3^Department of Health Management and Policy, College for Public Health and Social Justice, Saint Louis University, St. Louis, MO, United States; ^4^Department of Business Analytics, Business School, University of Colorado Denver, Denver, CO, United States; ^5^Office of Human Resources, Hubei Open University, Wuhan, China

**Keywords:** health care, industrial convergence, public health, geospatial, China

## Abstract

**Objective:**

This paper examines the impact of healthcare industry convergence on the performance of the public health system in the eastern, central, and western regions of China.

**Methods:**

Public health performance was measured by a composite index of three standards: average life expectancy at birth, perinatal mortality, and maternal mortality. The healthcare industry convergence was measured using a coupling coordination degree method. The spatial lag, spatial error, and spatial Durbin models were used to estimate the effect of healthcare industry convergence on public health system performance and this effect’s spatial dependence and heterogeneity across eastern, central, and western China using panel data from 30 Chinese provinces from 2002 to 2019.

**Results:**

The convergence of the healthcare industry significantly promotes regional public health [ β =0.576, 95% CI: (0.331,0.821)]. However, the convergence does not have a spatial spillover effect on the public health system at the national level. Additionally, analysis of regional heterogeneity shows that the direct effects of healthcare industry convergence on public health are positive and statistically significant for Eastern China, statistically insignificant for Central China, and positive and statistically significant for Western China. The indirect effects are negative, statistically significant, positive, statistically significant, and statistically insignificant for these three regions, respectively.

**Conclusion:**

Policy efforts should strengthen the convergence between the healthcare industry and relevant industries. It can produce more current healthcare services to improve public health and reduce regional health inequality.

## 1. Introduction

Healthcare industry convergence refers to the integration and collaboration between various sectors within the healthcare industry, such as medical services, healthcare delivery, pharmaceuticals, biotechnology, and information technology (1, 2). In China, the impact of healthcare industry convergence on public health can be significant. For example, convergence allows for the seamless flow of information among different sectors (3). It facilitates the use of advanced technologies, leading to better diagnosis, treatment, and monitoring of diseases, ultimately improving the overall quality of healthcare (4). Healthcare industry convergence also contributes to public health by emphasizing preventive care and health education. Through integrated platforms, individuals can access personalized health information, receive reminders for screenings and vaccinations, and engage in self-management of chronic conditions (5). This proactive approach can help reduce the disease burden, promote healthy lifestyles, and improve Chinese people’s health. Moreover, by integrating various sectors, such as telemedicine, mobile health applications, and e-commerce platforms, people in remote areas or those with limited mobility can receive timely medical consultations, access medication, and obtain health information more easily (6). Therefore, healthcare industry convergence helps in reducing regional disparities in healthcare access in China.

Although the benefits the healthcare industry convergence can bring to the public health system in China are extensive, the empirical study of healthcare industry convergence is few, especially the impact on public health conducted in a geospatial setting.

The concept of industry convergence earliest originated in Rosenberg’s work in the 1960s, which explained how different types of technologies in a common field eventually developed into the machine tool industry (7). It can be defined narrowly as the merging of sectors formed by the same technological convergence or broadly as the convergence of various industrial fields of economic development (8). In contrast, others described it as the dissolution of knowledge boundaries between specific industries leading to the merging of scientific knowledge (9). However, industrial convergence can occur not only at the technological or knowledge level, but also in the industrial value chain, where it leads to market convergence based on the diversified demand of consumers (10). With the new era of information and communication technology (ICT), industrial information integration was proposed in 2005 to focus on the digital and information transformation integration of industrial sectors (11). Above all, we can trace that the definition of industry integration has been enriching with social development. In a word, industry convergence is a phenomenon in which the boundaries between two or more different economic sectors become blurred from common convergence foundation (12).

With the increase of health needs and aging, more and more scholars have had a heated discussion on the integration and development of healthcare industry.

The popular integration application between medical areas and scientific technology innovation has attracted the attention of some scholars. Yin et al. discuss the prevention of COVID-19 from the perspective of industrial information integration (III), which was evaluated with gravity model and social network analysis to set up the industrial information integration of industrial sectors (13). At results, they propose an integrated framework for managing public health crises like COVID-19, which involves the fusion of big data intelligence with emergency management and scientific research. Saheb and Izadi identified the integration innovation mechanism between the IoT Big Data Analytics paradigm (IoTBDA) and healthcare services with a literature review of 46 papers on IoTBDA and 84 papers on healthcare industry (14). Their study shows the convergence of LoTBDA and healthcare industry has triggered the novel health applications and systems. Eidam et al. used a methodology of industry/technology sector classification based on publication and patent data (15). They proposed that the trend of Ubiquitous healthcare is an expression of an overall industry convergence between the ICT and health sector. Lee and Lee concluded that contactless medical services based on IT have gained a renewed significance during three time periods: pre-,during-,and post-COVID-19 (16). It is evident that these literature mainly emphasize the role of ICT in the healthcare industry, and overlook the integration characteristics of healthcare industry and ICT.

In addition to this, there are also a series of integration research about the development of healthcare industry and relevant service industries.

An emerging field is the convergence between healthcare service and tourism industries, known as wellness tourism industry (17). Zhang et al. proposed an improved Markov chain combination forecasting method to evaluate the development of healthcare tourism industry (18). They also used various processing methods to study the impact of medical tourism industry on regional economic performance economic results. Qian et al. utilized the input–output method to measure the convergence degree of the health tourism industry in China, and analyzed the driving factors with a multiple linear regression model (19). Majeed and Kim believe that this convergence can expand and extend the traditional medical and tourism industry chain beyond traditional boundaries, improving residents’ life satisfaction, health conditions, and mental health (20). The integration of sports and health industries has been a new paradigm for high-quality economic development (21). Chen explores the construction of guarantee conditions, incubation platforms and the synergy between supply and demand for the development of the integrated sports and health industry based on the big data (22). Xu et al. used coupling and coordination degree model to evaluate the convergence development degree between sports industry and health service industry (23). The health and care service integration is a new care service mode for the older adult (24). Chen et al. construct the utility model to explore how to allocate resource between pension and medical institutions in the current mode of integrating health and care services for older people (25). In the context of healthy aging in China, some scholars claim that the goal of integrating health and care services is to provide more reliable care and medical protection for the healthy life of the older adult (26). Most of them focus on the qualitative analysis of operation mechanism. There is only a few to measure the convergence level and the analysis of influencing factors. But there is no quantitative analysis of the impact of this convergence on health outcome.

To sum up, existing literature has analyzed several specific integration industries within healthcare industry. In fact, the integrating development of healthcare industry is not limited to above industries. To our knowledge, there is also lack of a comprehensive measurement and analysis for healthcare industry convergence. Furthermore, most of them did not conduct further quantitative analysis of the industry integration on public health. Research about spatial spillover is also relatively rare.

To fill the gap in the literature, this study systematically measures the level of integration of the healthcare industry in China based on data from 30 provinces, and uses spatial econometric models to explore the impact of the development of healthcare integration on public health and the differences between the eastern, central, and western regions.

## 2. Materials and methods

### 2.1. Data

To investigate the relationship between the convergence development of the healthcare industry and public health, this study utilized data from 30 provinces in inland China from 2002 to 2019. The data were collected from various sources, including the China Statistical Yearbook, China Health Statistical Yearbook, China Industrial Statistical Yearbook, China Statistical Yearbook of the Tertiary Industry, and China Statistical Yearbook on the High Technology Industry. Tibet was excluded from the study due to data unavailability. The data were collected annually to understand the trends over time comprehensively.

### 2.2. Survey measures

#### 2.2.1. Public health performance

In general, it is more challenging to distinguish regional differences in public health from individual health. Given the limitations of a single index evaluation and data availability, this paper uses a comprehensive indicator of average life expectancy, perinatal mortality, and maternal mortality to measure public health performance (27, 28). The aggregate health outcome indicator is specified as follows.


(1) $$Health_{it}=w_{1}LE_{it}+w_{2}PM_{it}+w_{3}MM_{it}$$

Where $Health_{it}$ is public health outcomes, $LE_{it}$ is average life expectancy at birth, $PM_{it}$ the perinatal mortality, and $MM_{it}$ is the maternal mortality. Three objective variable weights $w_{1},w_{2},w_{3}$ are calculated with the entropy method (29) (Appendix A discusses the technical details).

This study uses linear dimensionless functions to standardize three indicators. Formula (2) for average life expectancy and Formula (3) for the other two negative indicators are as follows.


(2) $$p_{it}=\frac{y_{it}-\min\left(y_{it}\right)}{\max\left(y_{it}\right)-\min\left(y_{it}\right)}$$

(3) $$p_{it}^{\prime }=\frac{\max\left(y_{it}\right)-y_{it}}{\max\left(y_{it}\right)-\min\left(y_{it}\right)}$$

Where $y_{it}$ is the initial value of the indicator y of a province i ( i=1,2,⋯,n ) in year t , $p_{it}$ , and $p_{it}^{\prime }$ are the standardized values, $\min\left(y_{it}\right)$ is the minimum value of indicator y in all provinces in year t , $\max\left(y_{it}\right)$ is the maximum value of the indicator y in all provinces in year t .

Finally, the health value ranges from 0 to 1. That is, the higher the health value is, the better the health level of the population is.

#### 2.2.2. Convergence degree of the healthcare industry

The 2019 China Health Industry Statistical Classification proposed a series of new businesses relevant to healthcare services, which are considered the outcome of industry convergence. This study defines industry convergence as the process through which the healthcare industry is extended as the boundaries between the traditional healthcare industry (H0) and relevant industries (H1-H12) become less clear (30). Table 1 lists industries under the national industries classification (GB/T 4754–2017).

**Table 1:** Industries according to National industries classification (GB/T 4754–2017).

Code	Industry
H0	Healthcare service
H1	Electricity, heat, gas and water production and supply
H2	Manufacturing
H3	Transportation, storage and postal services
H4	Information transmission, software and information technology services
H5	Wholesale and retail trade
H6	Accommodation and catering services
H7	Financial services
H8	Scientific research and technology services
H9	Water conservancy, environment and public facilities management
H10	Education industry
H11	Culture, sports and entertainment industry
H12	Public administration, social security and social organizations

To understand the convergence in the overall healthcare industry (Appendix B discusses the technical details), we use employment number and investment in fixed assets as two input variables. We also use gross domestic product (GDP), or the number of people served, as one output variable. We measure the degree of industry convergence between the healthcare industry (H0) and 12 relevant industries(H1-H12) by coupling the coordination degree model and entropy method (As Table 2 shows). Furthermore, we synthesize 12 industry convergence degrees to generate the whole convergence degree for the healthcare industry at the province level in China (Table 3 shows).

**Table 2:** Average convergence degree between healthcare service industry and other 12 industries for 30 provinces from 2002 to 2019.

Province	H1	H2	H3	H4	H5	H6	H7	H8	H9	H10	H11	H12
Beijing	0.35	0.28	0.33	0.45	0.42	0.43	0.40	0.44	0.36	0.40	0.42	0.31
Tianjin	0.22	0.25	0.26	0.25	0.28	0.24	0.26	0.26	0.26	0.28	0.22	0.21
Hebei	0.49	0.48	0.50	0.39	0.47	0.43	0.53	0.41	0.51	0.56	0.49	0.50
Shanxi	0.35	0.29	0.36	0.27	0.33	0.31	0.35	0.25	0.37	0.39	0.35	0.35
*Inner Mongolia*	0.35	0.27	0.33	0.24	0.28	0.29	0.33	0.24	0.34	0.32	0.31	0.32
Liaoning	0.40	0.40	0.44	0.44	0.42	0.41	0.45	0.38	0.46	0.48	0.40	0.42
Jilin	0.31	0.29	0.30	0.27	0.29	0.27	0.32	0.27	0.34	0.37	0.31	0.31
Heilongjiang	0.37	0.31	0.34	0.31	0.32	0.31	0.36	0.29	0.38	0.41	0.34	0.36
Shanghai	0.32	0.34	0.35	0.39	0.42	0.41	0.39	0.36	0.36	0.36	0.33	0.29
Jiangsu	0.55	0.61	0.58	0.60	0.58	0.55	0.59	0.53	0.60	0.62	0.54	0.55
Zhejiang	0.46	0.53	0.53	0.51	0.51	0.51	0.53	0.44	0.53	0.51	0.47	0.49
Anhui	0.40	0.37	0.44	0.34	0.38	0.38	0.43	0.34	0.43	0.47	0.40	0.41
Fujian	0.40	0.38	0.41	0.39	0.38	0.39	0.40	0.31	0.40	0.41	0.37	0.38
Jiangxi	0.39	0.35	0.38	0.30	0.33	0.36	0.38	0.28	0.39	0.45	0.37	0.39
Shandong	0.57	0.64	0.63	0.58	0.63	0.60	0.62	0.56	0.62	0.68	0.60	0.66
Henan	0.52	0.53	0.55	0.44	0.51	0.52	0.53	0.42	0.57	0.62	0.53	0.55
Hubei	0.45	0.41	0.46	0.40	0.43	0.44	0.45	0.39	0.49	0.53	0.44	0.46
Hunan	0.48	0.41	0.48	0.39	0.42	0.45	0.46	0.40	0.50	0.54	0.48	0.50
Guangdong	0.62	0.64	0.67	0.67	0.64	0.67	0.66	0.57	0.66	0.64	0.57	0.60
Guangxi	0.40	0.32	0.39	0.32	0.34	0.36	0.38	0.30	0.42	0.43	0.39	0.39
Hainan	0.13	0.11	0.16	0.13	0.14	0.20	0.15	0.11	0.17	0.16	0.16	0.14
Chongqing	0.33	0.28	0.34	0.31	0.30	0.33	0.32	0.27	0.34	0.36	0.30	0.30
Sichuan	0.59	0.45	0.55	0.51	0.46	0.51	0.53	0.42	0.56	0.60	0.52	0.56
Guizhou	0.30	0.24	0.32	0.24	0.26	0.28	0.29	0.22	0.32	0.34	0.30	0.31
Yunnan	0.36	0.28	0.36	0.29	0.33	0.34	0.35	0.26	0.37	0.39	0.37	0.38
Tibet	0.05	0.01	0.04	0.02	0.01	0.04	0.04	0.01	0.00	0.01	0.03	0.07
Shaanxi	0.37	0.33	0.38	0.36	0.36	0.38	0.38	0.35	0.41	0.47	0.39	0.40
Gansu	0.30	0.22	0.27	0.21	0.25	0.25	0.27	0.23	0.29	0.32	0.29	0.32
Qinghai	0.14	0.10	0.11	0.08	0.09	0.10	0.10	0.09	0.12	0.11	0.09	0.12
Ningxia	0.17	0.11	0.13	0.10	0.11	0.11	0.13	0.09	0.14	0.14	0.12	0.13
Xinjiang	0.37	0.24	0.29	0.24	0.26	0.25	0.30	0.22	0.31	0.31	0.33	0.32

H1: Electric power, heat, gas and water production and supply industry, H2: Manufacturing industry, H3: Transport, storage and post industry, H4: Information transmission, software and information technology services industry, H5: Wholesale and retail industry, H6: Accommodation and catering industry, H7: Financial industry, H8: Scientific research and technological services industry, H9: Water conservancy, environment and public facility management industry, H10: Education, H11: Culture, sports and entertainment industry, H12: Public administration, social security and social organization.

**Table 3:** Convergence degree of healthcare service industry (HICD) and rank for 30 provinces in China from 2002 to 2019.

Province	2002	2004	2006	2008	2010	2012	2014	2016	2018	2019	Total HICD	Rank
Guangdong	0.63	0.63	0.65	0.64	0.63	0.63	0.63	0.62	0.63	0.63	0.63	1
Shandong	0.60	0.59	0.61	0.62	0.62	0.63	0.62	0.63	0.61	0.61	0.62	2
Jiangsu	0.54	0.54	0.59	0.57	0.57	0.58	0.58	0.57	0.58	0.59	0.59	3
Henan	0.48	0.48	0.51	0.52	0.53	0.53	0.53	0.55	0.54	0.54	0.52	4
Sichuan	0.51	0.50	0.51	0.51	0.53	0.53	0.52	0.52	0.52	0.52	0.52	5
Zhejiang	0.49	0.51	0.52	0.51	0.49	0.51	0.50	0.51	0.50	0.50	0.50	6
Hebei	0.48	0.48	0.49	0.47	0.48	0.48	0.48	0.48	0.48	0.47	0.48	7
Hunan	0.43	0.43	0.44	0.44	0.46	0.46	0.47	0.48	0.47	0.48	0.46	8
Hubei	0.41	0.41	0.43	0.44	0.45	0.47	0.46	0.46	0.45	0.46	0.44	9
Liaoning	0.44	0.44	0.34	0.42	0.44	0.46	0.42	0.36	0.35	0.46	0.46	10
Anhui	0.36	0.36	0.38	0.40	0.41	0.41	0.41	0.41	0.41	0.41	0.40	11
Beijing	0.38	0.38	0.35	0.38	0.39	0.38	0.37	0.36	0.36	0.41	0.42	12
Fujian	0.39	0.37	0.38	0.37	0.38	0.39	0.38	0.39	0.38	0.39	0.38	13
Shanxi	0.35	0.34	0.37	0.37	0.38	0.40	0.40	0.40	0.40	0.40	0.38	14
Guangxi	0.35	0.35	0.36	0.36	0.38	0.38	0.38	0.38	0.37	0.37	0.37	15
Jiangxi	0.36	0.35	0.36	0.35	0.36	0.37	0.36	0.37	0.36	0.37	0.36	16
Shanghai	0.32	0.32	0.36	0.38	0.36	0.36	0.33	0.33	0.40	0.40	0.40	17
Heilongjiang	0.36	0.35	0.36	0.37	0.35	0.34	0.33	0.32	0.30	0.30	0.34	18
Yunnan	0.32	0.32	0.33	0.34	0.34	0.35	0.34	0.36	0.36	0.36	0.34	19
Shaanxi	0.33	0.32	0.33	0.33	0.35	0.34	0.32	0.33	0.31	0.31	0.33	20
Chongqing	0.29	0.29	0.31	0.31	0.32	0.32	0.32	0.33	0.32	0.32	0.31	21
Jilin	0.30	0.30	0.33	0.33	0.31	0.31	0.29	0.30	0.28	0.28	0.30	22
*Inner Mongolia*	0.29	0.29	0.31	0.32	0.32	0.31	0.31	0.29	0.28	0.28	0.30	23
Guizhou	0.26	0.26	0.26	0.26	0.27	0.28	0.29	0.31	0.32	0.33	0.28	24
Xinjiang	0.30	0.29	0.30	0.28	0.27	0.28	0.28	0.28	0.28	0.29	0.28	25
Gansu	0.26	0.25	0.27	0.26	0.27	0.27	0.27	0.28	0.26	0.26	0.27	26
Tianjin	0.26	0.26	0.26	0.26	0.24	0.27	0.25	0.24	0.22	0.22	0.25	27
Hainan	0.14	0.14	0.14	0.14	0.15	0.14	0.13	0.14	0.14	0.15	0.14	28
Ningxia	0.11	0.12	0.13	0.13	0.12	0.12	0.12	0.12	0.13	0.14	0.12	29
Qinghai	0.09	0.09	0.10	0.10	0.10	0.11	0.10	0.11	0.10	0.11	0.10	30

Total HICD is the average of HICD from 2002 to 2019.

#### 2.2.3. Covariates

According to existing research, other variables may also affect public health performance. This paper controls for the following variables in the model:

1. Aging ratio, represented by the proportion of the total population aged over 65 years in area i in year t ( $Age_{it}$ ). Aging is an important indicator of public health performance (31).
2. *Per capita* years of education, represented by different years of education of the population in area i in year t ( $Edu_{it}$ ). *Per capita* education years are calculated by the average years of education for primary school graduation (6 years), junior high school graduation (9 years), senior high school graduation (12 years), and bachelor’s education (16 years). The higher education years people have, the more ways and channels they have to improve their health (31).
3. Real *per capita* GDP, represented by the economic level in area i in year t ( $RGDP_{it}$ ), is used as a measure of economic growth. The real *per capita* GDP eliminates the influence of price with GDP deflator (the base year is 2002).
4. Urbanization ratio, represented by the proportion of the urban population in area i in year t ( $UBR_{it}$ ). Urbanization is usually accompanied by population migration to the cities, improving the health level of residents mainly through increased medical resources, and perfecting the medical insurance system and public health infrastructure investment (32).
5. Ratio of health expenditure to a total financial budget, represented by the proportion of local government health expenditures in the total financial budget in area i in year t ( $GOV_{it}$ ).When the government spends more on health, it results in greater accessibility and convenience of medical services for the local residents.

### 2.3. Statistical modeling

In this study, we aim to analyze the impact of the convergence development of the healthcare industry on public health performance at the provincial level. To adequately address the spatial dependencies and potential spillover effects, we employ a spatial econometric model instead of panel models in most studies.

#### 2.3.1. Spatial correlation test

It is necessary to examine significant spatial correlations in public health before conducting spatial econometric models (33). We use Global Moran’s I to explore the spatial distribution of public health performance; the formula is shown in Equation (4),


(4) $$Moran^{'}sI=\frac{\sum_{i=1}^{n}\sum_{j=1}^{n}w_{ij}\left(x_{i}-\bar{x}\right)\left(x_{j}-\bar{x}\right)}{S^{2}\sum_{i=1}^{n}\sum_{j=1}^{n}w_{ij}}$$

Where $S^{2}$ and $\bar{x}$ are calculated by the following equations:


$$S^{2}=\frac{1}{n}\overset{n}{\underset{i=1}{\sum }}{\left(x_{i}-\bar{x}\right)}^{2}$$

$$\bar{x}=\frac{1}{n}\overset{n}{\underset{i=1}{\sum }}x_{i}$$

In the equation, $x_{i}$ is the public health performance metrics of regional observations, n is the number of regions.

This paper utilizes a distance-based weight matrix known as the geographical adjacency weight matrix W . In this matrix, a value of 1 means that regions i and j are spatially adjacent, and 0 means that regions i and j are not spatially adjacent:


$$W_{ij}=\{\begin{array}{c} 1,i\;isadjacentto\;j;\\ 0,i\;isn^{\prime }t\;adjacentto\;j. \end{array}\left(i\neq j\right)$$

In addition to global Moran’s I, we also use Local Moran’s I to test for spatial correlation in a specific local area. The formula for Local Moran’s is shown in equation (5).


(5) $$I_{i}=\frac{\left(x_{i}-\bar{x}\right)}{S^{2}}\overset{n}{\underset{i=1}{\sum }}w_{ij}\left(x_{j}-\bar{x}\right)\;$$

#### 2.3.2. Spatial econometric model

Based on the spatial correlation test, there are three common spatial econometric models: spatial lag model (SLM), spatial error model (SEM), and spatial Durbin model (SDM).

When the dependent variable is spatially correlated, the SLM is used with the following formula:


$$\begin{array}{c} \ln Health_{it}=\rho \overset{n}{\underset{j=1}{\sum }}w_{ij}\ln Health_{it}+\beta_{0}\ln HICD_{it}\\ +\beta \ln X_{it}+\mu_{i}+v_{t}+\varepsilon_{it} \end{array}$$

When the model concerns the spatial dependence reflected in the residuals, the SEM is used as following:


$$\ln Health_{it}=\beta_{0}\ln HICD_{it}+\beta \ln X_{it}+\lambda \overset{n}{\underset{j=1}{\sum }}w_{ij}\varepsilon_{t}+\mu_{i}+v_{t}+\gamma_{it}$$

When the spatial correlation is presented in both the dependent and independent variables, the SDM is used:


$$\begin{array}{l} \ln Health_{it}=\rho \overset{n}{\underset{j=1}{\sum }}w_{ij}\ln Health_{it}+\beta_{0}\ln HICD_{it}+\beta \ln X_{it}\\ +\theta_{1}\overset{n}{\underset{j=1}{\sum }}w_{ij}\ln HICD_{it}+\theta_{2}\overset{n}{\underset{j=1}{\sum }}w_{ij}\ln X_{it}+\mu_{i}+v_{t}+\varepsilon_{it} \end{array}$$

To reduce the effect of heteroskedasticity and data fluctuations on the model estimation results, the variables in the model are log-transformed. $Health_{it}$ denotes public health performance in area i at time t ; $HICD_{it}$ represents the convergence degree of the health industry; $X_{it}$ are the control variable vectors, including aging ratio $\left(Age_{it}\right)$ , *per capita* years of education $\;\left(Edu_{it}\right)$ , real *per capita* gross domestic product $\left(RGDP_{it}\right)$ , urbanization ratio ( $URB_{it}$ ), ratio of health expenditure to total financial expenditure ( $GOV_{it}$ ); ρ denotes the spatial autoregressive model; $w_{ij}$ is the element of spatial weight matrix; β is the regression coefficient of independent variable; λ represents the autocorrelation coefficient of residual error term; θ is the spatial lag coefficient of the independent variable; $\mu_{i}$ and $v_{t}$ are the individual effects and the time effects, respectively; $\varepsilon_{it}$ is the random error item; $\gamma_{it}$ is a new random error item $\;\left(\gamma \sim N\left(0,\sigma^{2}I_{n}\right)\right)$ .

## 3. Results

### 3.1. Descriptive statistics

Table 4 provides a statistical summary of all variables in the 30 provinces from 2002 to 2019. Table 5 summarizes the global Moran’s I for public health performance, which shows a consistent decreasing trend in recent years but remains statistically significant and positive. These results showed a significant spatial correlation in the public health performance of 30 provinces during the sample period.

**Table 4:** Statistical summary of all variables.

Variables	Variable explanation	Unit	Mean	Std.Dev	Min	Max
$Health_{it}$	Public health outcomes	Index	0.683	0.152	0	1
$LE_{it}$	Average life expectancy at birth	Year	74.976	3.312	65.49	84.04
$PM_{it}$	Perinatal mortality	‰	7.875	4.012	1.8	24.82
$MM_{it}$	Maternal mortality	One in 100,000	25.035	22.407	1.1	160.3
$HICD_{it}$	Convergence degree of healthcare service industry	Index	0.373	0.131	0.092	0.649
$Age_{it}$	Aging ratio	%	9.586	2.141	4.763	16.375
$Edu_{it}$	*Per capita* years of education	Year	8.68	1.041	6.04	12.86
$RGDP_{it}$	Real *per capita* gross domestic product	Yuan per person	28797.39	24461.17	325	154360.77
$URB_{it}$	Urbanization ratio	%	52.339	14.654	24.675	94.152
$GOV_{it}$	Ratio of health expenditure to total financial expenditure	%	5.436	1.98	1.47	9.647

Data source: Calculated by the authors.

**Table 5:** Moran’s I values of public health for 30 provinces in China during 2002–2019.

Year	Moran’s I	Z-value	*P*-value	Year	Moran’s I	Z-value	*P*-value
2002	0.335	3.253	0.001	2011	0.369	3.497	0.000
2003	0.332	3.192	0.001	2012	0.356	3.350	0.000
2004	0.393	3.694	0.000	2013	0.370	3.489	0.000
2005	0.404	3.796	0.000	2014	0.356	3.431	0.000
2006	0.414	3.829	0.000	2015	0.335	3.249	0.001
2007	0.398	3.692	0.000	2016	0.322	3.136	0.001
2008	0.379	3.536	0.000	2017	0.323	3.103	0.001
2009	0.385	3.582	0.000	2018	0.320	3.072	0.001
2010	0.366	3.428	0.000	2019	0.276	2.722	0.003

Data source: Calculated by the authors.

**Figure 1:**
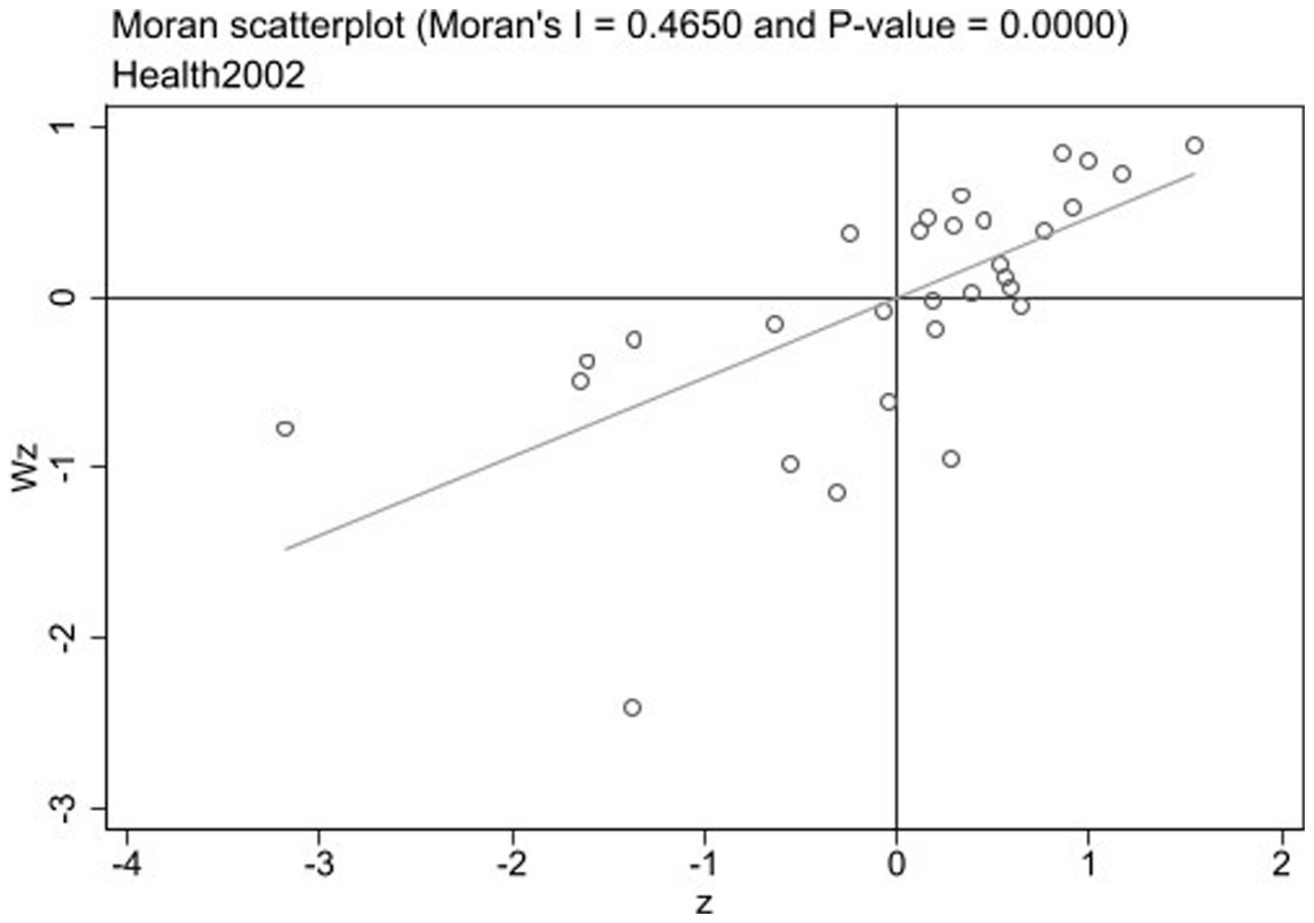
Local Moran’s I scatter plot of 30 provinces in 2002.

**Figure 2:**
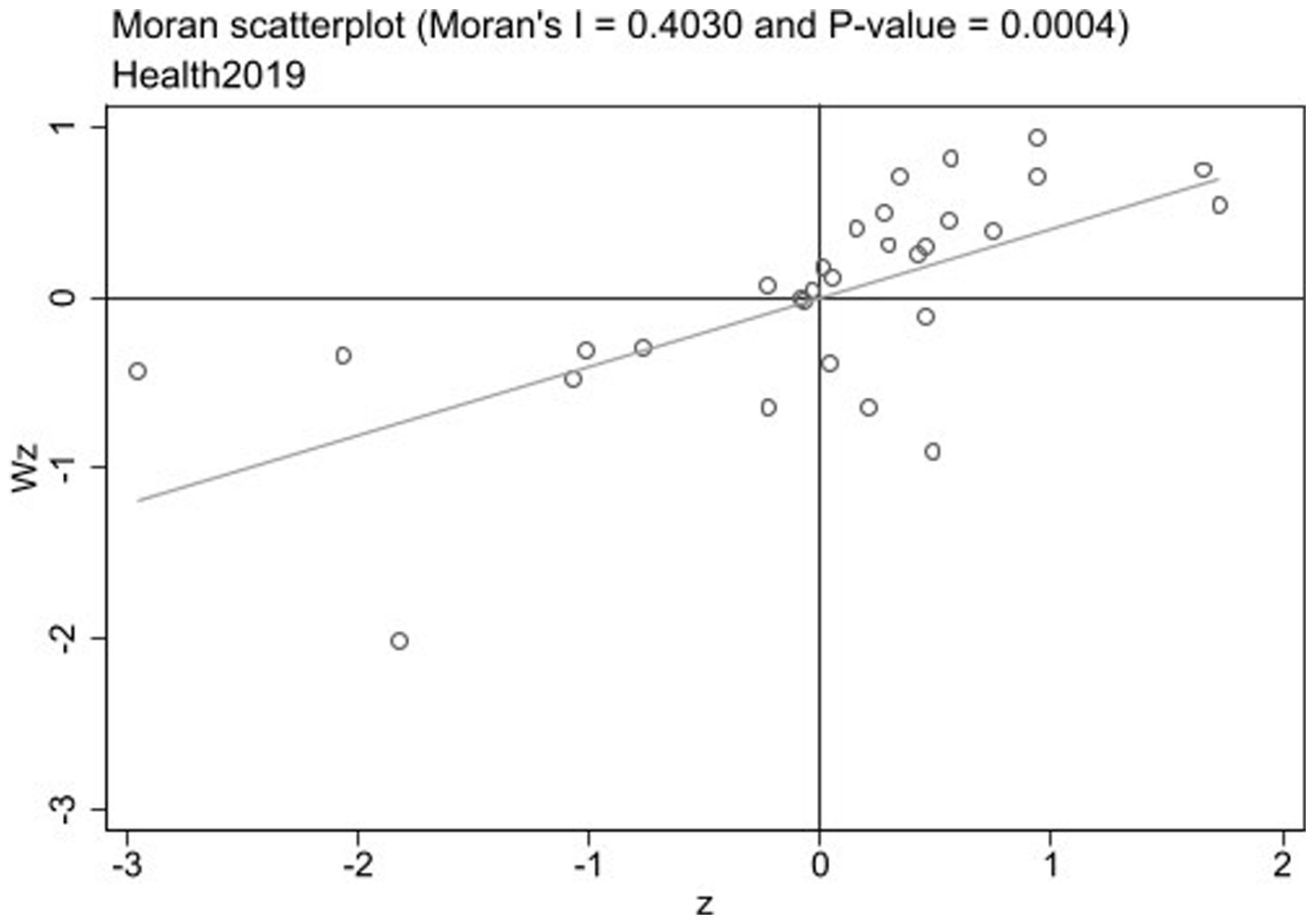
Local Moran’s I scatter plot of 30 provinces in 2019.

Local Moran’s I is a more accurate tool for analyzing the level of spatial clustering among provinces compared to global Moran’s I. Local Moran’s I scatter plots in 2002 and 2019 are shown in Figures 1, 2. The scatter plots are divided into four quadrants: quadrant I (high–high agglomeration) and quadrant III (low–low agglomeration) represent positive spatial correlations, and quadrant II (Low-High agglomeration) and quadrant IV (High-low agglomeration) represent negative spatial correlations. Figures 1, 2 show that most provinces fall in quadrants I and III, indicating a positive spatial correlation in public health across Chinese provinces. In 2002 and 2019, there are two-thirds of the provinces located in the high–high agglomeration quadrants, while the provinces in Midwestern China(Qinghai, Xinjiang, Yunnan, Guiyang, Gansu, Ningxia, Inner Mongolia), belong to the low–low agglomeration quadrant; these provinces are generally regarded as the poor economy and scarce public service resources (34).

### 3.2. Results of the impact of healthcare industry convergence on public health with spatial econometric model

Based on the results of Moran’s I, we used the spatial panel model to examine the factors affecting public health. When $\left(H_{0}:\theta =0\right)$ the likelihood ratio (LR) tests were conducted, the SAR $\left(50.33,\;\;p<0.05\right)$ and SEM $\left(48.96,\;\;p<0.05\right)$ were rejected against the SDM. This result implies that the SDM model outperforms the other two models and was chosen for regression analysis. LR tests were then conducted to determine the best model specification. The test results show the spatial-fixed and time-fixed effects $\left(402.17,\;\;p=0.00\right)$ were significant at the level of 1%. Therefore, it is reasonable to choose a model with two-way fixed effects. The Hausman test further confirms the conclusion (p<0.05) , which also rejected the random effects model specification $\left(34.53,\;\;p=0.00\right)$ . Therefore, these results indicate that the SDM with spatial and time-period fixed effects best fits our data.

Table 6 reports the results of four estimation methods (SDM, SLM, SEM, FE). The results suggest that the convergence development of the healthcare industry has a significant positive impact on public health performance. With regard to controlling variables, the results indicate that *per capita* years of education ( $\ln Edu_{it}$ ) and urbanization ( $\ln URB_{it}$ ) were positively associated with public health performance while aging ( $\ln Age_{it}$ ) has significantly negative associations.

**Table 6:** Four models about the impact of healthcare service industry convergence on public health in China.

Variables	SDM	SLM	SEM	FE
$\ln HICD_{it}$	0.576*** (4.61)	0.141** (2.48)	0.146** (2.57)	0.426*** (3.73)
$\ln Age_{it}$	−0.486*** (−6.67)	−0.34*** (−6.3)	−0.406*** (−6.69)	−0.279*** (−4.88)
$\ln Edu_{it}$	1.235*** (5.83)	0.827*** (4.48)	1.001*** (5.12)	0.888*** (4.38)
$\ln RGDP_{it}$	−0.229** (−2.44)	−0.071* (−1.9)	−0.091** (−2.29)	−0.036 (−0.86)
$\ln URB_{it}$	0.609*** (5.55)	0.609*** (6.91)	0.647*** (7.17)	0.552*** (5.01)
$\ln GOV_{it}$	−0.172*** (−4.48)	−0.144*** (−4.72)	−0.142*** (−4.42)	−0.186*** (−5.39)
$W\cdot \ln ICD_{it}$	−0.115 (−0.56)			
$W\cdot \ln Age_{it}$	0.381*** (3.51)			
$W\cdot \ln Edu_{it}$	−0.939*** (−3.11)			
$W\cdot \ln RGDP_{it}$	0.26** (2.31)			
$W\cdot \ln URB_{it}$	−0.315* (−1.8)			
$W\cdot \ln GOV_{it}$	0.021 (0.34)			
ρ	0.239*** (4.44)	0.167*** (3.26)		
λ			0.267*** (4.81)	
Log-likelihood	411.1338	327.7815	333.2948	
$R^{2}$	0.1891	0.4675	0.4888	0.2744

The regression coefficients are outside the parentheses, and t-values are inside the parentheses; *, **, and *** indicate statistical significance at the levels of 10, 5, and 1% levels.

The coefficients of the SDM do not directly reflect the spillover effects of explanatory variables (X) on the dependent variable (Y) , which can lead to potentially misleading conclusions. Thus, the partial differential is used to estimate further the direct and indirect effects of convergence development on the healthcare industry and those of other control variables on public health. Table 7 shows each variable’s direct, indirect and total effect on public health performance. We found that the core independent variable of $\ln HICD_{it}$ (0.153) has a direct impact on promoting overall public health. However, we did not observe a significant spatial spillover effects on neighboring regions. The results of the direct effects are consistent with the SDM empirical results. For the results of indirect effects, the coefficient of $\ln Edu_{it}$ (−1.076) was significantly negative, while $\ln Age_{it}$ (0.235) and $\ln RGDP_{it}$ (0.188) were significantly positive.

**Table 7:** The direct, indirect, and total effects of SDM in China.

Variables	Direct effect	Indirect effect	Total effect
$\ln HICD_{it}$	0.153*** (2.61)	−0.020 (−0.19)	0.134 (1.09)
$\ln Age_{it}$	−0.528*** (−8.07)	0.235** (2.05)	−0.294*** (−2.9)
$\ln Edu_{it}$	1.132*** (6.24)	−1.076*** (−3.39)	0.055 (0.16)
$\ln RGDP_{it}$	−0.185*** (−3.01)	0.188** (1.98)	0.003 (0.04)
$\ln URB_{it}$	0.745*** (7.95)	−0.053 (−0.28)	0.692*** (3.65)
$\ln GOV_{it}$	−0.150*** (−4.24)	−0.008 (−0.12)	−0.158** (−2.5)

The regression coefficients are outside the parentheses, and t-values are inside the parentheses; *, **, and *** indicate statistical significance at the levels of 10, 5, and 1% levels.

### 3.3. Analysis of empirical results in east, central and west regions

We then divided the 30 sample provinces into the eastern, central, and western regions according to their geographical location and economic development. The SDM model is again used to estimate the association between the convergence development of the healthcare industry and public health performance. The decomposition results of the direct, indirect and total effects are shown in Table 8. For the core explanatory variable $\left(\ln ICD_{it}\right)$ , the total effect is significant in the eastern region (−0.442) and central region (1.278), but not in the western region. The direct effects are significant positive in the eastern region (0.438) and western region (0.445), while the indirect effects are significantly negative in the eastern region (−0.880) and positive in the central region (1.490). These results indicate that the relationship between the convergence development of the healthcare industry and public health performance varied significantly among the three regions. We also find that other controlling variables’ impacts on public health differed for the eastern, central and western regions of China.

**Table 8:** The direct, indirect, and total effects of SDM for the Eastern, central, and Western region.

Variables	The Eastern Region			The Central Region			The Western Region		
	Direct effect	Indirect effect	Total effect	Direct effect	Indirect effect	Total effect	Direct effect	Indirect effect	Total effect
$\ln HICD_{it}$	0.438*** (3.02)	−0.880*** (−3.59)	−0.442* (−1.88)	−0.212 (−0.53)	1.490*** (3.66)	1.278*** (4.04)	0.445** (2.06)	−0.747 (−1.62)	−0.302 (−0.51)
$\ln Age_{it}$	−0.234*** (−3.23)	0.201*** (2.33)	−0.033 (−0.56)	−0.740*** (−5.06)	0.623*** (3.81)	−0.117 (−0.94)	−0.338** (−2.43)	0.012 (0.05)	−0.326 (−1.2)
$\ln Edu_{it}$	−0.207 (−0.69)	0.236 (0.64)	0.028 (0.09)	0.635* (1.84)	−0.342 (−0.85)	0.293 (0.72)	1.484*** (4.94)	−1.604*** (−3.13)	−0.120 (−0.20)
$\ln RGDP_{it}$	−0.328** (−2.4)	0.530*** (3.75)	0.203*** (3.58)	−0.343 (−1.34)	0.151 (0.49)	−0.193 (−1.43)	−0.495*** (−2.74)	1.115*** (3.57)	0.620*** (2.66)
$\ln URB_{it}$	−0.036 (−0.21)	−0.649*** (−3.82)	−0.685*** (−3.88)	0.329* (1.74)	0.266 (1.05)	0.594** (2.16)	0.517** (2.00)	−1.515** (−2.45)	−0.998 (−1.34)
$\ln GOV_{it}$	−0.073 (−1.41)	−0.037 (−0.55)	−0.110** (−2.11)	0.128* (1.69)	−0.250*** (−2.86)	−0.122 (−1.41)	−0.160*** (−2.89)	0.083 (0.77)	−0.077 (−0.64)

The eastern region was set to include Beijing, Tianjin, Hebei, Shandong, Liaoning, Shanghai, Zheijang, Jiangsu, Guangdong, Fujian, Hainan; the central region was set to include Heilongjiang, Jilin, Henan, Hubei, Hunan, Shanxi, Jiangxi, Anhui; the western region was set to include Gansu, Shaanxi, Sichuang, Chongqing, Guangxi, Guizhou, Inner Mongolia, Ningxia, Qinghai, Xinjiang, Yunnan. The regression coefficients are outside the parentheses, and t-values are inside the parentheses; *, **, and *** indicate statistical significance at the levels of 10, 5, and 1% levels.

## 4. Discussion

Our empirical analysis confirms the positive and significant effect of the convergence development of the healthcare industry on public health in China. Industry convergence can overturn traditional competitive logic and migrate beyond industry boundaries, thereby leading to changes in how value is distributed, created and captured (35, 36). In response to the speed of logical market changes and technological advances, many industries tend to produce products with short lead times, strong integration and low R&D costs and adopt cooperative competition (37). As industry convergence progresses, the boundaries of the healthcare industry have changed, giving rise to new business models, expanding the scope of current healthcare services, and providing a more comprehensive range of high-quality products and services to meet the health needs of the population (38). Eventually, realizing an enhanced industry value chain will improve public health (39). This study fills the gap in the literature on the association between the province-level convergence degree of the healthcare industry and public health.

Although the convergence development of the healthcare industry positively impacts public health, no spatial spillover effects have been noted in China. The reason might be that the healthcare industry has not yet fully penetrated relevant sectors, and the impact of industrial scale and brand have not yet been formed. Therefore, there is a need to improve the quality of convergence and deepen the cross-border integration of the healthcare industry to achieve better spillover effects on public health (40).

We have found a significant difference in the development of the healthcare industry in the east, central and western regions of China, which may be attributable to changes in regional economic development and industrial structure (41, 42). The eastern part has a more developed economy, talent pool, infrastructure, market environment and government support than China’s central and western regions. From a supply perspective, the convergence development of the healthcare industry is conducive to triggering innovation and generating new products that may increase industrial profits. From a demand perspective, residents in the eastern region have a substantial spending ability in the healthcare industry (43). Therefore, the higher integration of the healthcare industry in the east part has contributed to improving healthcare service capacity, directly affecting local public health. However, we have noted an imbalance in the allocation of policy resources, leading to a negative spatial spillover effect of the healthcare industry on public health in neighboring provinces (44).

The central and western regions have been less developed than the eastern region in China. However, the China government has implemented a series of effective supporting policies in recent years. The central region has been actively promoting the building of the Belt and Road Initiative, which aims to enhance communication and cooperation with neighboring areas (45). These efforts are expected to produce positive spatial spillover effects of the healthcare industry on public health in the central region. In the western region, all economic indicators have been improved due to the implementation of the Western Development Strategy. Economic growth has also promoted the convergence development of the healthcare industry, which has improved residents’ health service expenditures and health levels in the western region.

This study does have several limitations. Firstly, the analysis is based on the spatial Durbin model. Although SDM has been considering spatial autocorrelation, it may affect the accuracy of the results because the same spatial dependence relationships between provinces are assumed. Secondly, due to limited access to such information, we used province-level variables instead of city-level variables. Even though the convergence development of the healthcare industry and public health performance tend to be similar in a certain province, they are subject to minor differences for smaller cities within the same province based on local economic conditions and government policies. Thirdly, the period of this study is from 2002 to 2019 due to the lack of data. Therefore, it was not reflected in the association between the convergence development of the healthcare industry and public health during the COVID-19 pandemic from 2020 to 2022. Finally, this study is based on regional public health performance. We could not investigate the healthcare industry’s effect on individual health status. Future studies should link the regional development of the healthcare industry and individual health levels.

## 5. Conclusion

In conclusion, this study fills the gap in the literature on the impact of healthcare industry convergence on public health performance. Based on the panel data of 30 provinces in China from 2002 to 2019, the main findings of this study indicate a significantly positive direct association between the convergence development of the healthcare industry and public health. However, no spillover effect was observed in China. Moreover, the study found substantial variation in the impact of healthcare industry convergence among the eastern, central, and western regions due to differing social, economic, and medical development levels.

Building upon these findings enriches the theoretical research on public health by extending the convergence development of the healthcare industry. Policymakers should produce integrated platforms to offer more comprehensive and efficient care. Furthermore, they should also focus on regional differences to redistribute capital, policy, and human resources in the healthcare industry, thus improving public health and reducing health inequality between regions.

## Data availability statement

The original contributions presented in the study are included in the article/Supplementary material, further inquiries can be directed to the corresponding author.

## Author contributions

WL: conceptualization, data curation, and writing-original draft. KZ: methodology, software, and writing-original draft. EL: conceptualization, formal analysis, and writing-review and editing. WP: methodology, formal analysis, and writing-review and editing. CF: methodology, software, and writing-review and editing. QH: methodology, data curation, and writing-review and editing. LT: formal analysis, Writing-review and editing. All authors contributed to the article and approved the submitted version.

## Funding

The authors would like to acknowledge the financial support by the National Social Science Foundation of China (No. 20BTJ013) and First Class Discipline of Zhejiang-A (Zhejiang University of Finance and Economics-Statistics).

## Conflict of interest

The authors declare that the research was conducted in the absence of any commercial or financial relationships that could be construed as a potential conflict of interest.

## Publisher’s note

All claims expressed in this article are solely those of the authors and do not necessarily represent those of their affiliated organizations, or those of the publisher, the editors and the reviewers. Any product that may be evaluated in this article, or claim that may be made by its manufacturer, is not guaranteed or endorsed by the publisher.

## Supplementary material

The Supplementary material for this article can be found online at: https://www.frontiersin.org/articles/10.3389/fpubh.2023.1194375/full#supplementary-material
