# Supplementary material for: The impact of healthcare industry convergence on the performance of the public health system: a geospatial modeling study of provincial panel data from China
Source: Front Public Health. 2023 Sep 12;11:1194375. doi: 10.3389/fpubh.2023.1194375 (PMC10520712; doi:10.3389/fpubh.2023.1194375)
Supplement: Supplementary file 1 [file Data_sheet_1.pdf]

Appendix A. Entropy method for calculating three variable weights about public health

Entropy method is a kind of objective information weight method. We adopt entropy method to figure out the evaluation weight of the indicators. The steps involved were as follows.

First, standardize the initial positive indicator (average life expectancy) with Formula (A1) and two negative indicators (perinatal mortality and maternal mortality) with Formula (A2).

$$p_{ij} = \frac{y_{ij} - \min(y_j)}{\max(y_j) - \min(y_j)} \quad \text{(A1)}$$

$$p'_{ij} = \frac{\max(y_j) - y_{ij}}{\max(y_j) - \min(y_j)} \quad \text{(A2)}$$

Where y_{ij} is the initial value of indicator $j (j = 1,2,3)$ of province $i (i = 1,2,\cdots,30)$, p_{ij} or p'_{ij} is the standardized value, $\min(y_j)$ is the minimum value of indicator j in all provinces, $\max(y_j)$ is the maximum value of indicator j in all provinces.

Second, we calculate the entropy value with Formula (A3).

$$e_j = -k\sum_{i=1}^{n} p_{ij} \ln p_{ij} \, , k = 1/\ln(n) \quad \text{(A3)}$$

Then, the difference values are calculated with Formula (A4).

$$g_j = 1 - e_j \quad \text{(A4)}$$

Finally, we obtain the weights for average life expectancy (43%), perinatal mortality (35%), maternal mortality (22%) by means of Formula (A5).

$$w_j = g_j / \sum_{j=1}^{m} g_j \quad \text{(A5)}$$

Appendix B. Coupling Coordination Degree Model for calculating the convergence degree of healthcare industry at the level of province

The coupling coordination degree (CCD) model is a mathematical model used to quantify the degree of industrial convergence between two or more entities on the concept of coordination. This study uses 3 variables, including employment number and investment in fixed assets as two input variables, and GDP (for H1-H9 and H11) or number of people served (for H0,H10,H12) as one output variable.

Firstly, standardize three initial positive indicators (two input indicators and one output indicator) with Formula (B1).

$$p_{ij} = \frac{y_{ij} - \min(y_j)}{\max(y_j) - \min(y_j)} \quad \text{(B1)}$$

Where y_{ij} is the initial value of indicator $j(j = 1,2,3)$ of province $i(i = 1,2,\cdots,30)$, $p_{ij} \in [0,1]$ is the standardized value, $\min(y_j)$ is the minimum value of indicator j in all provinces, $\max(y_j)$ is the maximum value of indicator j in all provinces.

Secondly, calculate the comprehensive indicator to evaluate the industrial development level for 13 industries with Formula (B2).

$$U_k = \sum_{j=1}^{3} \omega_{kj} p_{kj} \quad \text{(B2)}$$

Where U_k is the comprehensive evaluation value for industry k ($k = 0,1,\cdots,12$) according to two input variables and one output variable. U_0 is the evaluation value for healthcare industry. $U_1, U_2, \cdots U_{12}$ are the evaluation values for other 12 relevant industries. ω_{kj} is the weight of indicator j for industry k from Entropy method.

Thirdly, we measure industrial convergence degree respectively between healthcare industry and other 12 relevant industries by coupling coordination degree model with Formula (B3) and (B4).A smaller value indicates a smaller convergence degree.

Formula B3 measures the coordination degree between healthcare industry and relevant industry.

$$C(U_0, U_k) = 2 \times \sqrt{U_0 \times U_k} \,/\, (U_0 + U_k) \quad \text{(B3)}$$

Where $C(U_0, U_k)$ is the coordination degree between U_0 and U_k.

Formula B4 furthermore measures the coupling coordination degree between U_0 and U_k to reflect the actual sustainable development.

$$D = \left\{ C \times (\alpha \times U_0 + \beta \times U_k) \right\}^{\frac{1}{2}} \quad \text{(B4)}$$

Where α=0.5 and β=0.5 represent the relative importance for healthcare industry and other relevant industry k .

Furthermore, we measure the overall degree of healthcare industrial convergence with coupling coordination degree (CCD). It is calculated as the weighted average of the coordination degree for 12 pairs of industries, where the weights represent the relative importance of the industries in the convergence development from entropy.
